# Accelerating the Detection of Bacteria in Food Using Artificial Intelligence and Optical Imaging

**DOI:** 10.1128/aem.01828-22

**Published:** 2022-12-19

**Authors:** Luyao Ma, Jiyoon Yi, Nicharee Wisuthiphaet, Mason Earles, Nitin Nitin

**Affiliations:** a Department of Food Science and Technology, University of California, Davis, California, USA; b Department of Biological and Agricultural Engineering, University of California, Davis, California, USA; c Department of Viticulture and Enology, University of California, Davis, California, USA; University of Naples Federico II

**Keywords:** foodborne pathogen, rapid detection, microcolony, multispecies classification, machine learning, microbial indicator

## Abstract

In assessing food microbial safety, the presence of Escherichia coli is a critical indicator of fecal contamination. However, conventional detection methods require the isolation of bacterial macrocolonies for biochemical or genetic characterization, which takes a few days and is labor-intensive. In this study, we show that the real-time object detection and classification algorithm You Only Look Once version 4 (YOLOv4) can accurately identify the presence of E. coli at the microcolony stage after a 3-h cultivation. Integrating with phase-contrast microscopic imaging, YOLOv4 discriminated E. coli from seven other common foodborne bacterial species with an average precision of 94%. This approach also enabled the rapid quantification of E. coli concentrations over 3 orders of magnitude with an *R*^2^ of 0.995. For romaine lettuce spiked with E. coli (10 to 10^3^ CFU/g), the trained YOLOv4 detector had a false-negative rate of less than 10%. This approach accelerates analysis and avoids manual result determination, which has the potential to be applied as a rapid and user-friendly bacterial sensing approach in food industries.

**IMPORTANCE** A simple, cost-effective, and rapid method is desired to identify potential pathogen contamination in food products and thus prevent foodborne illnesses and outbreaks. This study combined artificial intelligence (AI) and optical imaging to detect bacteria at the microcolony stage within 3 h of inoculation. This approach eliminates the need for time-consuming culture-based colony isolation and resource-intensive molecular approaches for bacterial identification. The approach developed in this study is broadly applicable for the identification of diverse bacterial species. In addition, this approach can be implemented in resource-limited areas, as it does not require expensive instruments and significantly trained human resources. This AI-assisted detection not only achieves high accuracy in bacterial classification but also provides the potential for automated bacterial detection, reducing labor workloads in food industries, environmental monitoring, and clinical settings.

## INTRODUCTION

Early detection of microbial contamination in food products is critically important for consumer safety and outbreak prevention. The consumption of contaminated foods was estimated to cause 550 million illnesses (almost 1 in 10 people) and 230,000 deaths worldwide (1). The ultimate goal of food manufacturers is to detect pathogens before releasing their products to the market, preferably within hours after being processed. However, standard culture-based detection methods take a few days, by which time the foods likely have been distributed through the supply chain and consumed. Besides health concerns, the early detection of pathogens also significantly reduces the business costs associated with food recalls and liability (2). Despite the progress made in recent years, there are still gaps in the early detection of contaminated food products.

One of the main challenges in the early detection of microbial contamination is that many current methods require a relatively long cultivation time before bacterial characterization. The gold standard method involves pre-enrichment, enrichment, and colony isolation on various types of growth media to increase the number of targeted bacteria and partially inhibit the interference of background microbiota (3). The entire analysis could take 5 to 7 days, which is too long for the food industry, as many products have short shelf lives (3). Progress has been made to reduce the detection time and improve detection limits. For example, alternative culture-based methods, such as chromogenic-based methods and membrane filter methods, require 30 h to 48 h for bacterial colonies to grow (4, 5). To further reduce the turnaround time, a variety of molecular techniques have also been developed, including nucleic acid-based methods (e.g., PCR and whole-genome sequencing [WGS]), immunoassays (e.g., enzyme-linked immunoassay [ELISA]), and metabolic fingerprinting (vibrational spectroscopy, matrix-assisted laser desorption ionization–time of flight mass spectrometry [MALDI-TOF MS]) (3). However, most of these approaches (e.g., PCR, WGS, and MALDI-TOF MS) still require the enrichment and/or isolation of colonies. A few culture-independent approaches can detect foodborne pathogens within several hours after enrichment, but they do not yield bacterial isolates that are currently necessary for downstream characterization, such as serotyping and antimicrobial resistance profiling (6). In addition, these approaches require sophisticated equipment and specialized personnel, which are less accessible to food industries. Overall, current approaches do not fully meet the desired needs for a rapid, user-friendly, affordable, and nondestructive detection method, representing a technological gap in food safety.

One potential approach to reduce detection time in culture-based methods is to shorten the cultivation and analysis of bacterial colonies. Before forming visible macrocolonies (i.e., diameter at the millimeter level), bacteria generate a microcolony structure within a few division cycles. Instead of targeting the macrocolonies that require 16 to 48 h to form, a microcolony-level detection will significantly reduce the turnaround time to a few hours. Recently, a few studies have attempted to classify bacterial species based on the chemical properties of microcolonies. Chemical fingerprinting profiles of microcolonies were determined and classified within 6 to 24 h using vibrational spectroscopy, such as Raman spectroscopy and Fourier transform infrared (FT-IR) hyperspectral imaging (7–10). These spectroscopy-based methods are faster than traditional methods, but most of these studies lack quantitative reports of classification accuracy to detect target bacteria. The morphology of microcolonies also shows potential as a characteristic for bacterial differentiation. For example, different bacterial species show distinct microcolony phenotypes in terms of growth rate, ring count, and size (11). A multivariate analysis of microcolony images could classify Staphylococcus members at the species level with an accuracy of 98%, but this method required 11 h of incubation to reach a microcolony diameter up to 250 μm (12). Another study reduced the analysis to 6 h using forward-scattering imaging and the Bayes Network algorithm, but the genus-level classification rate was relatively low (i.e., 76.5%) (13). These studies indicate the opportunities to analyze the phenotypic properties of bacterial colonies at the early stages of cultivation, but some limitations need to be resolved to enable broader deployment of these methods. First, many food industries have access to standard light microscopes but typically are not equipped with more specialized systems, such as vibrational spectroscopy or other custom-made microscopes. Second, an ideal detection time should be less than a working shift period in the food industry (i.e., ~6 to 8 h). Third, to the best of our knowledge, no studies have been performed to identify bacteria in the presence of diverse bacterial species, which is a basic nature of food products. Lastly, more sophisticated data analysis, such as artificial intelligence (AI) approaches, can be applied to improve detection accuracy and sensitivity.

We evaluate the potential use of a rapid bacterial detection method using the object detection and classification algorithm You Only Look Once version 4 (YOLOv4) and wide-field white light optical microscopy. We targeted Escherichia coli as the indicator of fecal contamination (14). The approach does not require selective culture media and reduces the cultivation time to 3 h. Our method has the potential to be widely applied in food industries, environmental monitoring, and clinical settings and could aid in the rapid detection of bacteria.

## RESULTS

### Morphology of E. coli microcolonies.

**FIG 1:**
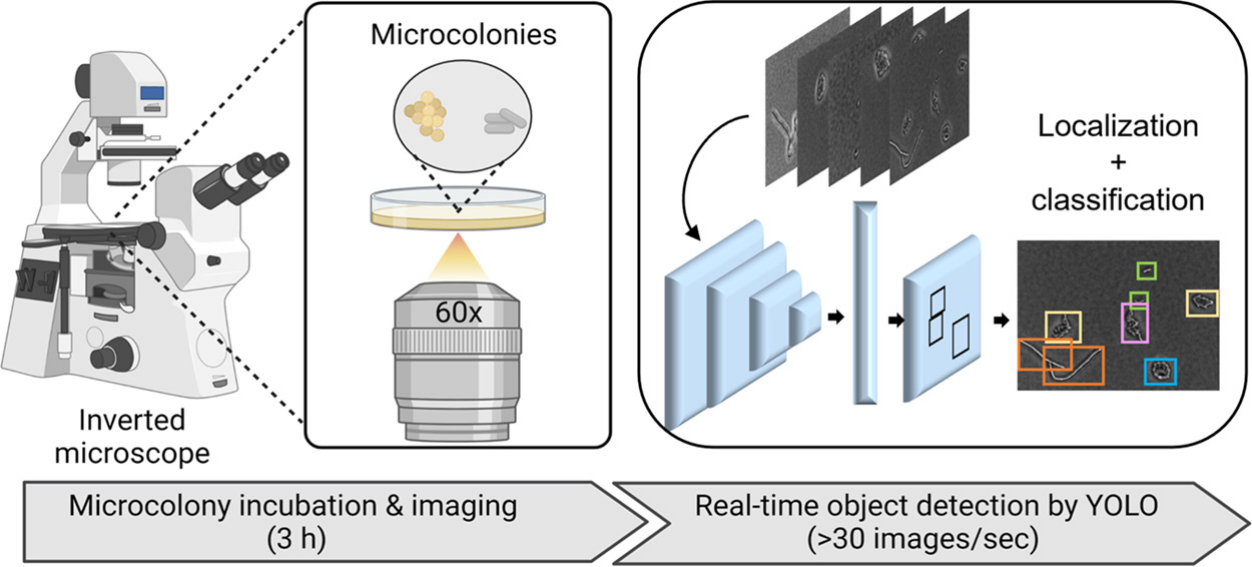
Workflow of YOLO-based microcolony classification. After a 3-h incubation, microcolonies were monitored using a phase-contrast microscope. The deep-learning algorithm You Only Look Once version 4 (YOLOv4) was applied to locate and classify the microcolonies. This figure was created with BioRender.com.

**FIG 2:**
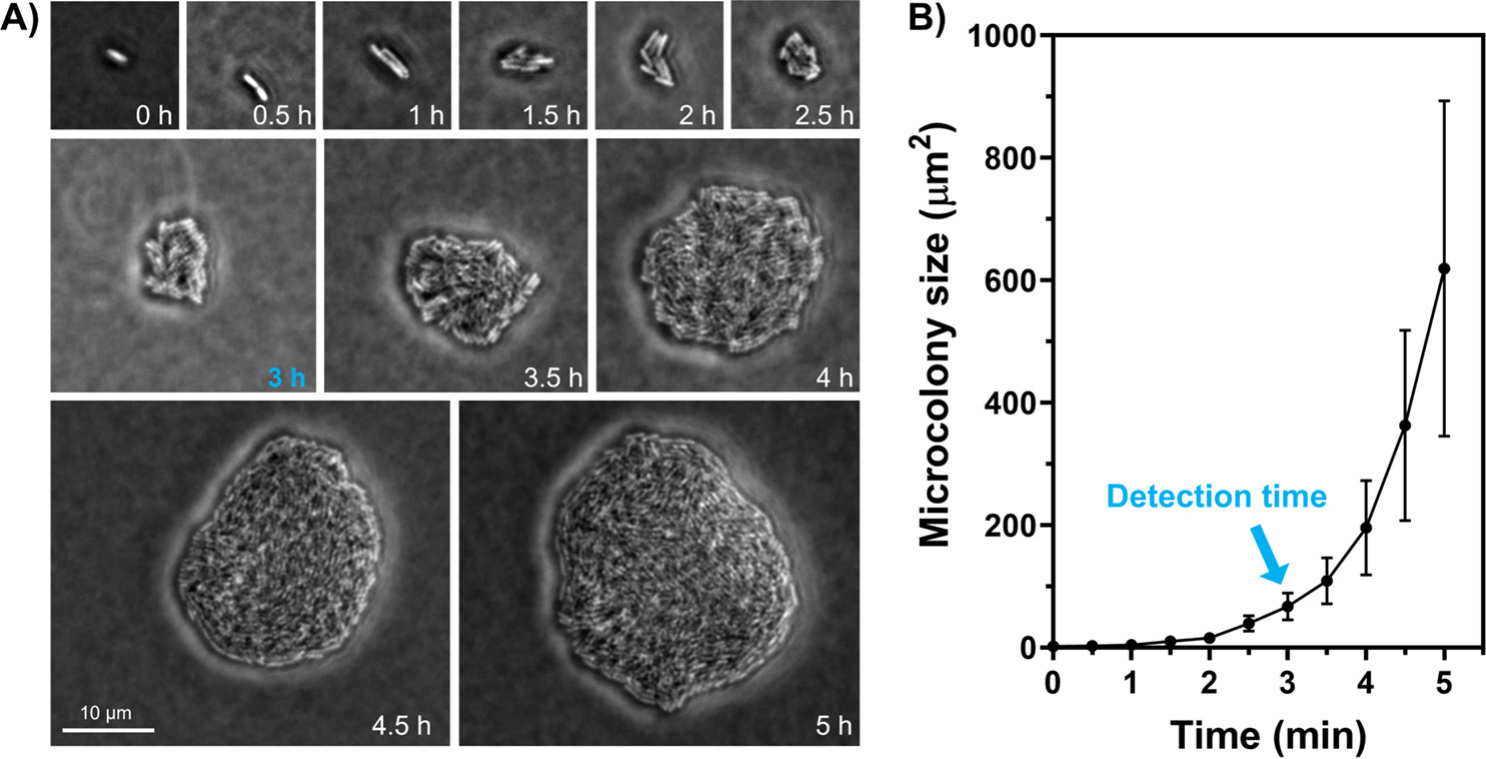
Growth profiles of E. coli microcolonies. (A) E. coli (LJH 1612) microcolonies were monitored under a phase-contrast microscope at various time points. (B) Averaged areas of E. coli microcolonies as a function of time. The results were obtained from 100 images for each time point. Error bars represent the standard deviations.

This study aims to develop a rapid bacterial detection method with a simple operation procedure using the standard bacterial cultivation and microscopic imaging commonly available in the food industry. The detection is based on an AI-enabled analysis of morphological differences among bacterial microcolonies. As shown in Fig. 1, our method consists of two steps, as follows: (i) microcolony incubation and white light imaging and (ii) real-time detection using the object detection and classification algorithm YOLOv4. To determine the appropriate incubation time for microcolony detection, we monitored the growth dynamics of E. coli using phase-contrast microscopy (Fig. 2). Figure 2A shows that a single bacterium attached to the agar surface and formed a microcolony consisting of thousand cells within 5 h. Several morphological changes occurred as the microcolonies grew. In the first two divisions, bacterial daughter cells were arranged in a 4-cell array (15). As cells grew longitudinally along a common axis, the microcolonies became elongated after 2 h of incubation. The anisotropic expansion might be due to the mechanical tensions between daughter cells, whose new poles are in contact and tend to elongate toward each other (16). Later, cells were pushed outward in all directions, generating a more isotropic and circular microcolony (i.e., 2.5 to 5 h). Meanwhile, we observed a dimensional transition from a monolayer to multiple layers after initiating the microcolony growth for ~3 h. The size of microcolonies increased in an exponential growth pattern (Fig. 2B). An average microcolony size of 66 μm^2^ was obtained at 3 h and increased by 10 times at 5 h of incubation (Fig. 2B). Other E. coli strains, such as the model strain K-12, followed a similar expansion trend (see Fig. S1 in the supplemental material). Taken together, we chose 3 h as the microcolony incubation time for two reasons. First, it was difficult to determine the focal plane of the microcolonies once three-dimensional (3D) structures are formed after 3 h. Second, microcolonies at 3 h had more biomass and complex cell arrangement patterns than in the earlier stage, which may provide more features for bacterial classification.

### Quantitative analysis of bacterial concentration using microcolony imaging.

**FIG 3:**
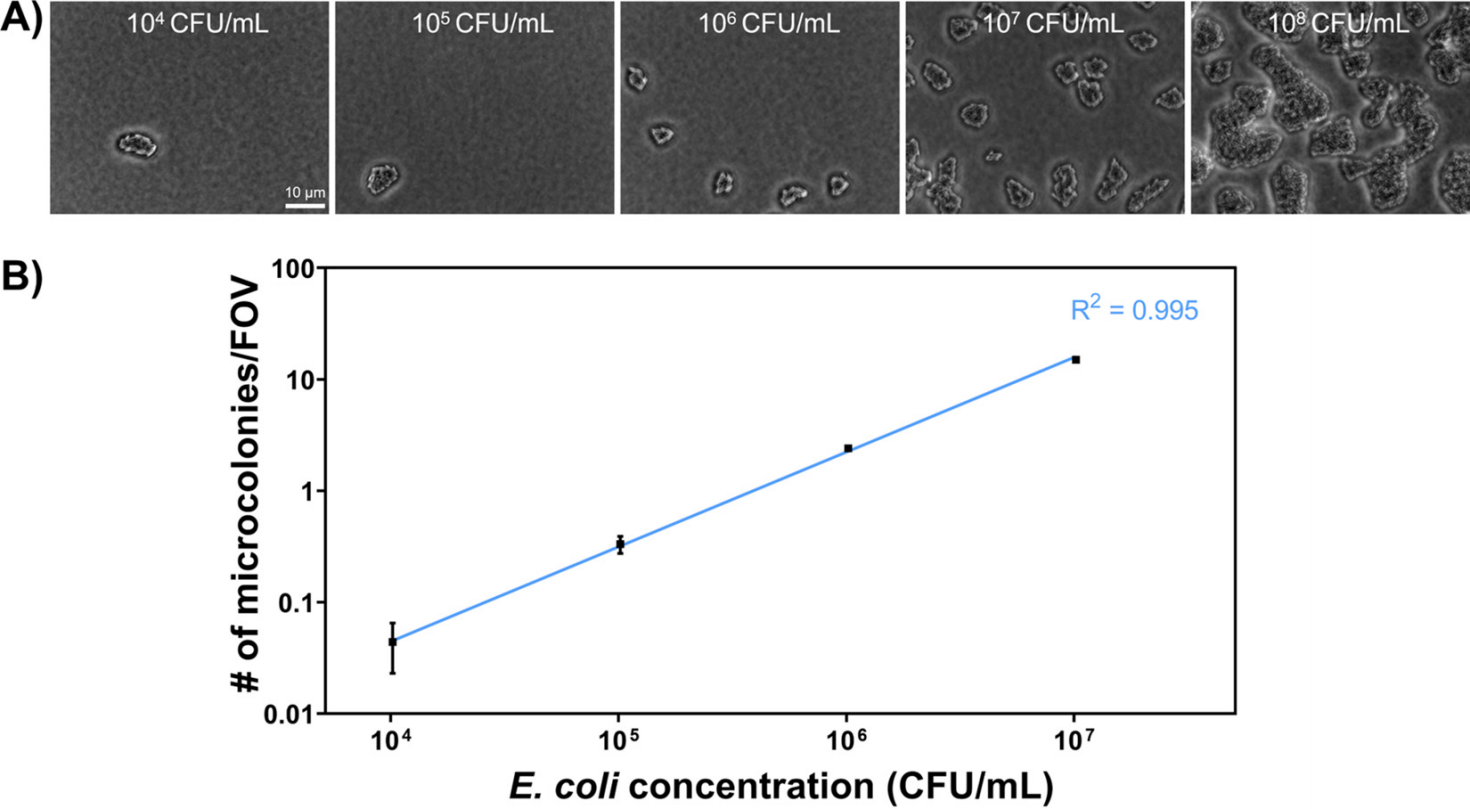
Determination of E. coli concentrations. (A) Representative microscopic images of E. coli (LJH 1612) microcolonies at a range of initial concentrations from 10^4^ to 10^8^ CFU/mL. (B) Initial E. coli concentrations versus the number of microcolonies per field of view (FOV). The solid line represents a linear regression. The results were calculated from 100 and 500 images for 10^6^ to 10^7^ CFU/mL and 10^4^ to 10^5^ CFU/mL, respectively. Error bars represent the standard deviations.

Enumeration of E. coli is performed routinely to indicate fecal pathogen contamination or unsanitary food processing, according to the Bacterial Analytical Manual established by the U.S. Food and Drug Administration (17). To investigate if our method is feasible for quantitative analysis, we counted the numbers of microcolonies per field of view (FOV; 72 μm by 55 μm). Briefly, a 10-fold serial dilution of E. coli LJH 1612 culture was inoculated onto soft tryptic soy agar (TSA) and then incubated at 37°C for 3 h. As indicated in Fig. 3A, bacterial microcolonies were randomly distributed on the surface of agar media. Microcolonies remained separate when the initial concentration was lower than 10^7^ CFU/mL and started merging at the concentration of 10^8^ CFU/mL. A linear regression (*R*^2^ = 0.995) was established between the initial E. coli concentration and the number of microcolonies in the FOV (Fig. 3B). Therefore, the initial concentration of E. coli could be estimated based on the number of microcolonies under microscopy in the range of 10^4^ to 10^7^ CFU/mL.

### Multispecies classification with YOLOv4.

**FIG 4:**
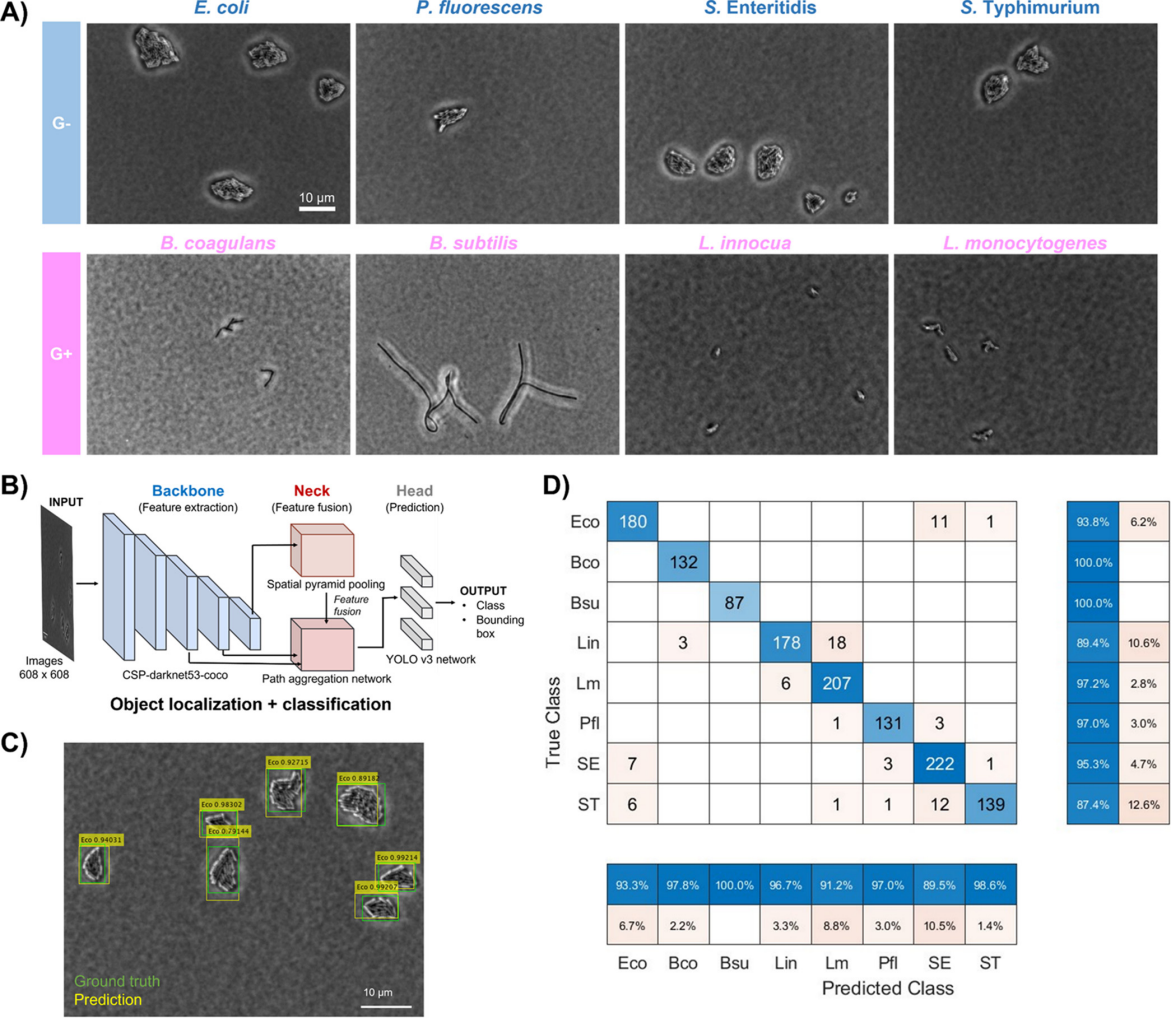
Microcolony detection and classification using YOLOv4. (A) Representative bacterial microcolonies of eight different species. In addition to E. coli, three Gram-negative bacteria (Pseudomonas fluorescens, Salmonella Enteritidis, and Salmonella Typhimurium) and four Gram-positive bacteria (Bacillus coagulans, Bacillus subtilis, Listeria innocua, and Listeria monocytogenes) were selected to represent the common spoilage and pathogenic bacterial species in the agri-food system. (B) The architecture of YOLOv4. (C) Example of YOLOv4 detection results. (D) Confusion matrix for microcolony classification of E. coli and other common spoilage and pathogenic bacterial species. The bottom and right panel report the precision and recall values of YOLOv4 in the test data set, respectively. The tested bacterial species included E. coli (Eco), *B. cogulans* (Bco), *B. sublitis* (Bsu), *L. innocua* (Lin), L. monocytogenes (Lm), P. fluorescens (Pfl), *S*. Enteritidis (SE), and *S.* Typhimurium (ST). For each species, 315 images were used for training (60%), validation (10%), and testing (30%).

We explored whether YOLOv4 is capable of distinguishing E. coli microcolony features from other spoilage and pathogenic bacteria in food products. To do so, we built the data set to represent diverse foodborne spoilage and pathogenic bacteria, including four Gram-negative species and four Gram-positive species. Specifically, Salmonella enterica (serotypes Enteritidis and Typhimurium) (18, 19) and Listeria monocytogenes (20) are regarded as the leading causes of foodborne outbreaks, while Pseudomonas fluorescens (21, 22), *Bacillus* spp. (Bacillus coagulans and Bacillus subtilis) (23), and Listeria innocua (24) are predominant in food processing environments and/or responsible for food spoilage. E. coli is often selected as an indicator of microbial contamination. The microcolonies from these bacterial species were imaged by phase-contrast microscopy (Fig. 4A). While there were distinct differences between the Gram-positive and Gram-negative microcolonies in terms of shape, size, and cell arrangement, the microcolonies of E. coli resembled those of other Gram-negative strains.

The advantage of YOLOv4 is its high speed. With YOLOv4, one can achieve real-time object detection above the human perception of 30 frames per second. The architecture of YOLOv4 consists of three parts (Fig. 4B), as follows: (i) CSP-darknet53-coco as a backbone neural network to extract microcolony features, (ii) a head to predict the location and classes of objects, and (iii) a neck between backbone and head to collect feature maps from different stages (25). As a result, YOLOv4 integrates the entire object detection and classification process in a single step (Fig. 4C), achieving real-time detection and identification (26).

We used YOLOv4 to classify E. coli and the other seven non-E. coli species. Considering the potential strain variation, we included six E. coli strains in the data set, which were isolated from foods, environments, animals, and humans (see Table S1 in the supplemental material). These isolation sources represented all potential transmission routes of foodborne bacteria from farms to tables. The data set was split into 60%, 10%, and 30% for training, validation, and testing of YOLOv4, respectively. The intersection over unit (IoU) was 0.78, indicating a good localization agreement between ground truth and predicted bounding boxes. As an evaluation metric for object detection, the mean average precision (mAP) was calculated over all classes (27). The mAP value was 0.94 (see Table S2 in the supplemental material), suggesting a good object detection performance. To assess the classification performance, we constructed the confusion matrix as shown in Fig. 4D. E. coli was discriminated successfully from other bacterial species, with a precision of 93.3%. The false results were misclassified mainly as Salmonella spp. In addition, the tested spoilage and pathogenic bacterial species were identified with high precision (89.5% to 100%) and high recall (87.4% to 100%).

Furthermore, we investigated whether YOLOv4 is able to distinguish E. coli microcolonies at the strain level. The data set was built from the images of six E. coli strains that were listed in Table S1. As expected, the microcolonies of these E. coli strains show high similarity and are visually indistinguishable (see Fig. S2A in the supplemental material). However, YOLOv4 was able to classify these E. coli strains with precision ranging from 87.3% to 97.2% (Fig. S2B). Therefore, a strain-level classification was possible using YOLOv4.

### Detection of E. coli as the contamination indicator in mixed bacterial culture.

**FIG 5:**
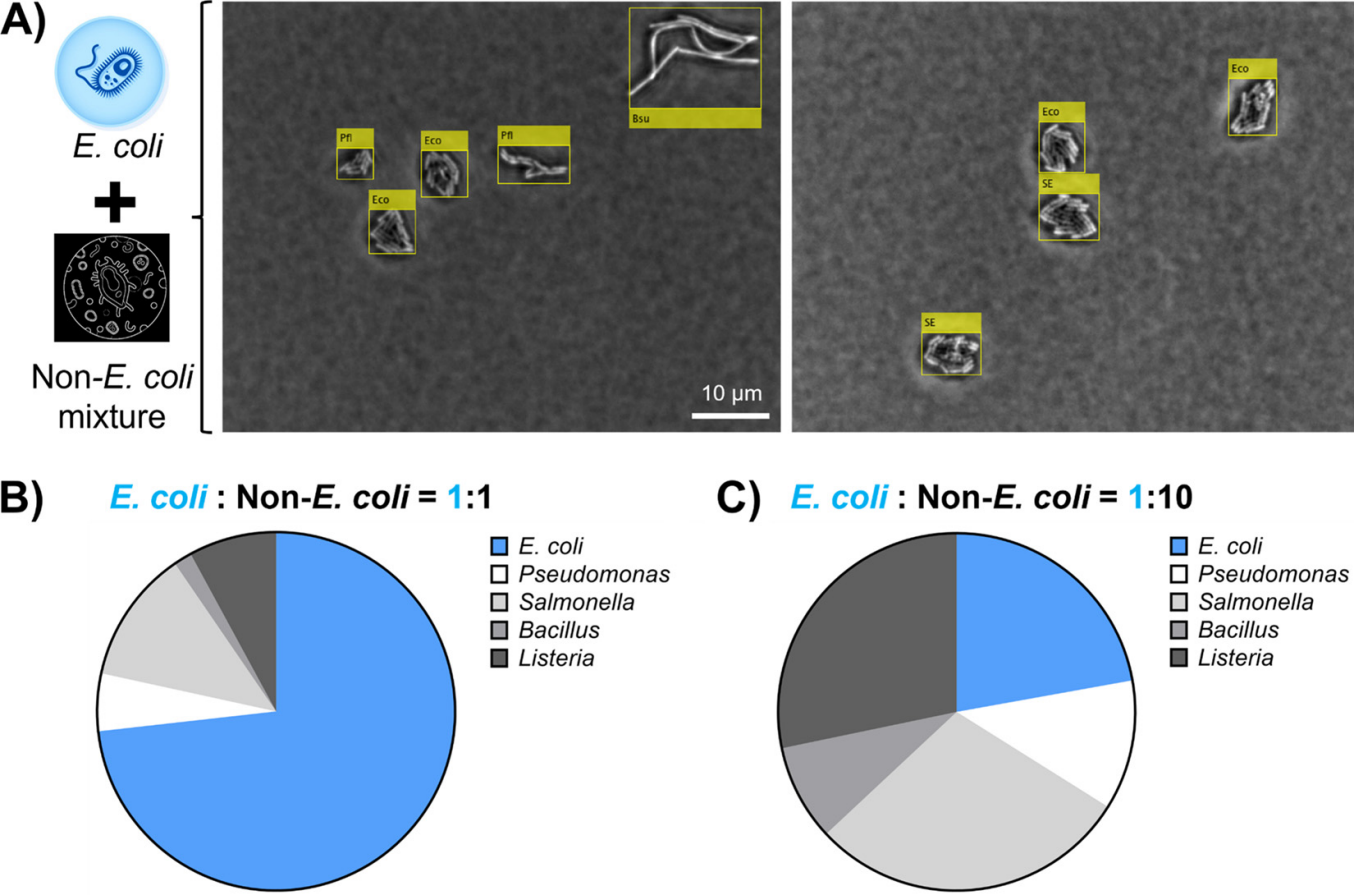
Detection of E. coli as a contamination indicator in mixed bacterial culture. Various ratios of E. coli and non-E. coli strains (i.e., Salmonella, Pseudomonas, *Bacillus*, and *Listeria* spp.) were mixed in phosphate-buffered saline, followed by microcolony detection and classification using the YOLOv4 detector. (A) Representative images were obtained from the mixture of E. coli and non-E. coli using phase-contrast microscopy. The bounding box and predicted bacterial species were determined using the YOLOv4 detector. (B) Composition percentages of E. coli and non-E. coli in the bacterial mixture determined by the YOLOv4 detector. The actual ratio of E. coli and non-E. coli during initial inoculation on agar was 1:1. (C) Composition percentages of E. coli and non-E. coli in the bacterial mixture determined by the YOLOv4 detector. The actual ratio of E. coli and non-E. coli during initial inoculation on agar was 1:10.

We further challenged the trained YOLOv4 detector by mixing E. coli and multiple bacterial species to generate a bacterial cocktail, mimicking the diverse microbiota in food products. The trained YOLOv4 detector was used to locate and classify microcolonies in the images (Fig. 5A). We mixed E. coli with Pseudomonas, Salmonella, *Bacillus*, and *Listeria* bacteria, which are found commonly in food products as spoilage and pathogenic bacteria. As shown in Fig. 5B, the ratio of E. coli and non-E. coli was predicted to be 2.69:1 when the actual initial ratio was 1:1. Even though YOLOv4 detector slightly overestimated the percentage of E. coli in the mixed culture, the predicted concentration difference between E. coli and non-E. coli was only 0.43 log CFU/mL (data not shown). When the initial concentration of E. coli decreased by 10 times, the YOLOv4 detector reported a 7.8-fold reduction in the ratio of E. coli and non-E. coli mixture (Fig. 5C). Overall, these results suggest a reasonably accurate semiquantification of E. coli in the complex bacterial culture.

### Detection of E. coli in fresh produce.

To evaluate the performance of the YOLOv4 detector in identifying E. coli in fresh produce, romaine lettuce was used as a food model, as it has been frequently reported as the vehicle of E. coli outbreaks. To recover bacteria, lettuce leaves were rinsed and homogenized in phosphate-buffered saline (PBS) using a blender. The rinse solution was deposited onto soft TSA plates and incubated at 37°C for 3 h. The detection of E. coli was performed using microcolony imaging and the YOLOv4 detector trained with the multiple bacterial species shown in Fig. 4. To validate the results from the YOLOv4 detector, a conventional plating assay was also conducted on selective sorbitol MacConkey agar. E. coli LJH 1612 was spiked on lettuce samples to mimic the low (i.e., ~10 CFU/g) and high levels (i.e., ~10^3^ CFU/g) of contamination, which was the contamination range frequently found in lettuce at retail locations (28, 29). As shown in Table 1, 11 out of 12 samples were identified correctly as E. coli positive (91.6%).

**TABLE 1:** Detection of E. coli^*a*^ on romaine lettuce using YOLOv4

Type of samples	E. coli concentration (log CFU/g lettuce)	
	Actual	Predicted^*b*^
Low E. coli load (*n* = 6)	1.38	2.95 ± 0.08
	1.20	2.18 ± 0.27
	0.90	2.43 ± 0.30
	1.09	Not detected
	0.45	1.70 ± 0.17
	1.47	2.10 ± 0.35
High E. coli load (*n* = 6)	3.37	3.46 ± 0.19
	3.39	3.43 ± 0.09
	3.29	2.30 ± 0.68
	2.51	3.06 ± 0.44
	2.75	3.16 ± 0.21
	2.60	2.99 ± 0.47

a *n* = 12.

b The value of predicted *E. coli* concentration is demonstrated as mean +/− standard deviation of triplicates.

## DISCUSSION

By integrating microscopy and machine learning, this study developed a platform to enable the rapid detection of E. coli within 3 h. Traditional culture-based methods rely on the isolation of bacterial single colonies for biochemical or genetic characterization (30). In contrast, YOLOv4-based imaging method detects bacteria by differentiating the features of microcolonies under a phase-contrast microscope (Fig. 1). Bacterial microcolonies show a wide spectrum of shapes that vary from circular to filamentous structures (16). This morphological difference was distinguishable visually between Gram-negative and Gram-positive bacteria but not within the tested Gram-negative species (Fig. 4A). Previous studies attempted to classify bacteria by calculating the light-scattering fingerprints of microcolonies, such as maximal growth rate, donutness, and energy density (12, 31). However, these studies were conducted using relatively larger microcolonies (~740 times larger area) than the current study and hence required a longer incubation time (11 to 20 h) than the current study (~3 h) for classification (12, 31). The previous studies illustrated the proof of concept for the microcolony-based detection method with manual feature selection and classification and achieved a detection accuracy of ~74% (12). This study uses a deep-learning algorithm to distinguish the microcolony features without requiring manual feature selection or prior knowledge (Fig. 4). A previous study has compared various deep-learning neural networks for counting macrocolonies, including two-stage (e.g., Faster R-CNN and Cascade R-CNN) and one-stage (e.g., YOLOv4 and EfficientDet-D2) algorithms (32). Among these algorithms, YOLOv4 showed the best detection performance and speed to count bacterial macrocolonies (32). In this study, YOLOv4 was further extended to classify bacteria at the microcolony stage for rapid detection, with a precision of over 90% at the genus, species, and strain levels (Fig. 4, Fig. S2).

The advantages of the YOLOv4-based detection method are multifold. First, this method provides a sample-to-answer analysis within 3 h, while the conventional culture-based method requires 1 to 7 days to complete tedious bacterial isolation and biochemical assays (3–5, 17). A few biochemical kits have been commercialized to shorten the analysis time, such as the API bacterial identification system (bioMérieux; ~20 min) and the Remel Micro-ID system (Thermo Scientific; a few hours). However, these commercial diagnostic kits still require the isolation of visible bacterial colonies, which is the most time-consuming step in bacterial detection (about 18 to 48 h) (3). Rather than detecting micrometer-scale colonies, this method targets microcolonies that are about 70 μm^2^, significantly reducing the incubation time. Alternatively, some molecular detection methods, such as real-time PCR, can directly detect E. coli from food homogenates without colony isolation. For example, After bacterial enrichment and DNA extraction, a real-time PCR method was able to detect E. coli at the concentration of 1 CFU/g, with a total analysis time of 9 h, including enrichment and sample preparation (33). This YOLOv4-based method also achieved a relatively low detection limit (~10 CFU/g) and reduced the analysis time to 3 h (Table 1). The detection sensitivity can be further improved by concentrating samples through centrifugation or filtration (34). Second, one of the potential advantages of this study is to simplify the detection protocol to microcolony incubation, imaging, and YOLOv4 detection. Conventional methods involve a set of procedures for bacterial enrichment and isolation on different nonselective and selective agar media, followed by microscopic confirmation (i.e., Gram staining) and biochemical assays. Colonies can also be classified using nucleic acid-based methods (e.g., PCR) but still require multiple steps for DNA extraction, purification, and amplification. This study has the potential to reduce the labor-intensive workflow by depositing bacterial samples onto agar media, imaging, and classifying bacteria *in situ*. Third, no selective agar was used in this study to specifically cultivate certain bacteria. Instead, a general-purpose growth medium (i.e., TSA) was used as the sole cultivation medium, reducing the laboratory labor and analysis cost. A previous study also reported the benefits of using nonselective media over selective agar due to the faster colony growth and more diverse bacterial species (35). Fourth, AI-based classification enables a more unbiased and accurate detection. Previously, analysts randomly pick presumptive colonies on selective agar media for species identification. This selection is subjective and only a limited number of colonies are tested (<10 out of hundreds or thousands), which is error prone and may generate false-negative results. Fifth, optical imaging is nondestructive on bacterial colonies, which can be reserved for further characterization, such as antimicrobial resistance and virulence. Last, this AI-based method has the potential to be cost-effective for food industries and resource-limited regions, as no sophisticated instrument is required besides a standard white light optical microscope. To further reduce the capital cost of a light microscope and to reduce the footprint of the microscope, future work may be focused on miniaturizing the imaging device (e.g., smartphone-based imaging and edge computing devices) for point-of-use detection of bacterial microcolonies. The automation of imaging acquisition is also expected to reduce the labor and time required for detection.

The results of this study are well-suited for various applications in food industries, environmental monitoring, and clinical settings. This study targeted E. coli as the critical indicator for bacterial contamination in food products. Following the quantitative analysis, a strong linear trend was observed between the actual and predicted E. coli concentrations (Fig. 3) (*R*^2^ = 0.995). YOLOv4 successfully classified E. coli in the presence of Salmonella, Pseudomonas, *Bacillus*, and *Listeria* bacteria (Fig. 5). According to the standardized risk management system (e.g., hazard analysis critical control point [HACCP]), the enumeration of E. coli is a key assessment to ensure good hygiene practices in food industries (17, 36). However, previous efforts focused only on either bacterial counting (37, 38) or classification (11, 13, 39). Our method bridges this technical gap, providing the identification and quantification of E. coli at once. Beyond E. coli detection, we anticipate that this method will be particularly useful for understanding the diversity of pathogens and commensal microbiota in food, water, and clinical samples. Future work can be performed to expand the current database to more bacterial species, especially foodborne pathogens (40). We expect it to provide complementary insights into the metagenomics data.

This study also evaluated the feasibility of using the trained YOLOv4 detector for the detection of E. coli in romaine lettuce, where diverse microbiota and food debris are present. The AI-based method successfully identified 11 out of 12 lettuce samples contaminated with E. coli (Table 1). This result suggests that this method has the potential to be applied for bacterial detection in a realistic and complex food matrix. To further evaluate the feasibility of the AI-based method, future studies may be focused on other food commodities that have different native microbiota and debris. The detection accuracy in food products can be improved by many approaches, such as removing food debris through sample filtration and collecting extra microcolony features through time-lapse imaging.

In conclusion, the results of this study suggest that the real-time object detection and classification algorithm YOLOv4 provides simple, fast, and accurate determination of E. coli contamination, which is used as a hygiene indicator microorganism in food industries. The detection targets bacterial microcolonies that are prepared with a short cultivation time under standardized conditions. With the aid of YOLOv4, bacterial classification can be completed instantly after the 3-h cultivation with high average precision (94%). The contamination of pathogenic and spoilage foodborne bacteria can be identified using E. coli as an indicator. This method also has the potential to classify multispecies bacterial cultures. The trained YOLOv4 detector successfully identified 11 out of 12 lettuce samples contaminated with E. coli, suggesting its potential application as a screening approach in food industries. Due to the relatively low equipment requirement and minimal hands-on operation, this method could be adapted by food industries and other resource-limited settings.

## MATERIALS AND METHODS

### Bacterial strains and routine cultivation.

A complete list of bacterial strains is summarized in Table S1. Six generic E. coli strains were tested to investigate the strain-level variation of microcolony morphology. These E. coli strains covered diverse isolation sources, including irrigation water (E. coli LJH 1612), soil (E. coli TVS 355), fresh produce (E. coli TVS 354), animal (E. coli K-12 and ATCC 35218), and clinical samples (E. coli ATCC 11775). To assess the taxonomical resolution of the microcolony-based method, we selected six non-E. coli strains from four different genera as representative strains, including Salmonella (*n* = 2), Pseudomonas (*n* = 1), *Bacillus* (*n* = 2), and *Listeria* (*n* = 2).

Bacterial strains were preserved at −80°C using glycerol as a cytoprotectant. To routinely prepare bacterial culture, the bacterial glycerol stock was streaked onto tryptic soy agar (TSA; 1.5% agarose, wt/vol) and incubated for 24 h. A single macrocolony was transferred from the TSA plate to tryptic soy broth, followed by shaking at 175 rpm overnight. The bacterial culture was incubated at 37°C for all strains except Pseudomonas fluorescens, which was incubated at 30°C instead. The fresh overnight culture was diluted in phosphate-buffered saline (PBS) to achieve defined concentrations for the microcolony study.

### Microcolony imaging by phase-contrast microscopy.

To form microcolonies, 1 μL of bacterial culture was deposited onto soft TSA plates (0.7% agarose, wt/vol) and incubated at 37°C for 3 h. The thickness of soft TSA plates was controlled to be ~1 mm by adding 2 mL of growth media into a 60-mm petri dish. Bacterial microcolonies on agar plates were observed directly in phase contrast mode using an Olympus X71 inverted microscope with a 60×/0.7 Ph2 Air objective (Olympus LUCPlan FL N). The white light source was provided by an Olympus TH4-100 lamp. Digital images (672 by 512 pixels with a pixel size of 107.5 nm) were acquired by an ORCA-ER digital camera (Hamamatsu, Japan).

### Optimization of microcolony growth time.

We assessed the appropriate incubation time to obtain sufficient morphological information on microcolonies, which is the main feature of bacterial classification. Briefly, E. coli overnight culture was adjusted to ~10^6^ CFU/mL, and 1 μL of aliquots was dropped onto freshly prepared soft TSA plates (0.7% agarose, wt/vol). As a result, ~1,000 microcolonies were formed in the spot zone with a diameter of ~3 mm, avoiding the aggregation of microcolonies at the early growth stage. Bacterial samples were incubated at 37°C. Images of microcolonies were acquired every 30 min for up to 5 h using the phase-contrast microscope. For each time point, 100 images were obtained to calculate the average and standard deviation of the microcolony sizes. The determination of microcolony size was performed using MATLAB 2022a (The MathWorks, USA). To segment microcolony clusters from the agar background, we normalized the intensity of images and converted the images to a binary scale. The total pixel numbers of each microcolony cluster were counted and reported as the microcolony size.

### Training YOLOv4 to classify multiple bacterial objects.

YOLOv4 for microcolony identification and classification was carried out using MATLAB computer vision toolbox model for YOLOv4 object detection on a computer equipped with a 14-core central processing unit (CPU; Intel E5-2682 v4) and a graphics processing unit (GPU; NVIDIA Quadro P6000 24GB). The data set contained 315 images for each bacterial strain, which were collected from 9 replicate samples in 3 independent experiments. First, we labeled all microcolonies in the data set using the MATLAB image labeler. Each bacterial species was annotated as a different class. Bounding boxes were assigned to microcolonies, providing both the class and location information. Images were shuffled randomly and split into a training data set (60%), a validation data set (10%), and a test data set (30%). The input image was resized from 672 by 512 pixels to 608 by 608 pixels. To improve training accuracy, data augmentation was performed on the training set with random horizontal flip, vertical flip, and rotation. CSP-darknet53-coco was used as the network backbone for feature extraction. This detector was pretrained on the COCO data set that consists of 80 different object categories. The pretrained weights were used as a starting point for transfer learning to increase the rate of convergence. We specified the network training options as follows: initial learning rate = 0.0002, mini-batch size = 4, and epochs = 200. The validation set was used to select the best parameters of the YOLOv4 detector during training.

After the training was completed, we evaluated the performance of the YOLOv4 detector using the test set. The evaluation measures included the intersection of over union (IoU), precision, recall, and mean average precision (mAP) (26, 27), as follows:

$$\mathrm{IoU}=\frac{P\cap T}{P\cup T}$$

$$\mathrm{Precision}=\frac{\mathrm{TP}}{\mathrm{TP}+\mathrm{FP}}$$

$$\mathrm{Recall}=\frac{\mathrm{TP}}{\mathrm{TP}+\mathrm{FN}}$$

$$\mathrm{mAP}=\frac{1}{n}\overset{k=n}{\underset{k=1}{\sum }}AP_{k}$$

$$AP_{k}=\overset{k=n-1}{\underset{k=0}{\sum }}\left[\mathrm{recall}\left(k\right)-\mathrm{recall}\;\left(k+1\right)\right]\times \mathrm{precision}(k)$$

where *P* is the area of the predicted bounding box and *T* is the area of the ground truth box for object localization. *TP*, *FP*, and *FN* represent true positive, false positive, and false negative, respectively. For *mAP*, *n* is the number of classes, and *AP_k_* is the average precision of class *k* and calculated as the area value under the precision-recall curve (41).

### Detection of E. coli in the presence of mixed bacterial culture.

E. coli is used commonly as an indicator of multispecies bacterial contamination. We prepared a mixed culture of E. coli LJH 1612 and non-E. coli strains to mimic the presence of spoilage and pathogenic bacteria in food systems. The following two scenarios were created to test our trained YOLOv4 detector: (i) equal initial concentration of E. coli and non-E. coli (E. coli: non-E. coli, 1:1) and a (ii) higher load of non-E. coli contamination (E. coli: non-E. coli, 1:10). P. fluorescens, *S.* Typhimurium, Bacillus subtilis, and Listeria monocytogenes listed in Table S1 were selected as the representative non-E. coli strains. For the first scenario (E. coli: non-E. coli, 1:1), an overnight culture of non-E. coli strains was separately prepared and diluted by 1,000 times in PBS. An equal volume of each non-E. coli bacterial culture was mixed to generate the non-E. coli cocktail. The overnight culture of E. coli LJH 1612 was diluted by 1,000 times in PBS. The same volume of E. coli and non-E. coli cocktail was mixed. For the second scenario (E. coli: non-E. coli, 1:10), the overnight culture of E. coli LJH 1612 was diluted by 10,000 times and non-E. coli by 1,000 times in PBS, followed by mixing the equal volume of E. coli and non-E. coli cocktail.

One microliter of the bacterial mixture was dropped onto freshly prepared soft TSA plates. Bacterial samples were incubated at 37°C for 3 h, followed by image acquisition using phase-contrast microscopy. Around 150 images were obtained per sample. The YOLOv4 detector was applied to locate, classify, and count microcolonies from different species. To validate the ratio of each strain, a plating assay was performed to enumerate individual bacterial overnight cultures.

### Detection of E. coli in food samples.

Romaine lettuce samples (*n* = 12) were purchased from grocery stores in Davis, California, between June and July 2022. The samples were transferred to the laboratory and analyzed within 1 h. Given that the prevalence and contamination level of E. coli in lettuce might be low (28, 42), we spiked the lettuce samples with a low load of E. coli (*n* = 6) and a high load of E. coli (*n* = 6). Briefly, 25 g of lettuce leaves were spiked with 10^2^ or 10^4^ CFU of E. coli LJH 1612 culture. After drying for 15 min, each sample was transferred into a sterile stomacher bag. To recover bacteria from the lettuce leaves, all samples were diluted in 50 mL of PBS and homogenized in a Stomacher 400 Circulator lab blender (Seward, USA) at 300 rpm for 3 min. One microliter of lettuce rinse was deposited onto soft TSA plates and incubated at 37°C for 3 h for microcolony growth. Three technical replicates were performed for each lettuce sample, and 100 images were obtained for each replicate. The YOLOv4 detector was applied to enumerate E. coli counts. In parallel, a conventional plating assay was applied to validate the results. In this process, the lettuce rinse was streaked onto the selective medium sorbitol MacConkey agar and incubated at 37°C for 24 h, followed by counting the presumptive E. coli colonies (43).
