# Supplementary material for: Accelerating the Detection of Bacteria in Food Using Artificial Intelligence and Optical Imaging
Source: Appl Environ Microbiol. 2022 Dec 19;89(1):e01828-22. doi: 10.1128/aem.01828-22 (PMC9888199; doi:10.1128/aem.01828-22)
Supplement: Supplemental file 1 — Supplemental material. Download aem.01828-22-s0001.pdf, PDF file, 0.3 MB [file aem.01828-22-s0001.pdf]

SUPPLEMENTARY MATERIALS

Accelerating detection of bacteria in food using AI and optical imaging

Luyao Ma[1], Jiyoon Yi[2], Nicharee Wisuthiphaet[1], Mason Earles[2,3], Nitin Nitin[1,2,*]

[1] Department of Food Science & Technology, University of California, Davis, CA 95616

[2] Department of Biological & Agricultural Engineering, University of California, Davis, CA 95616

[3] Department of Viticulture & Enology, University of California, Davis, CA 95616

*Corresponding Author: N. Nitin; Email: nnitin@ucdavis.edu

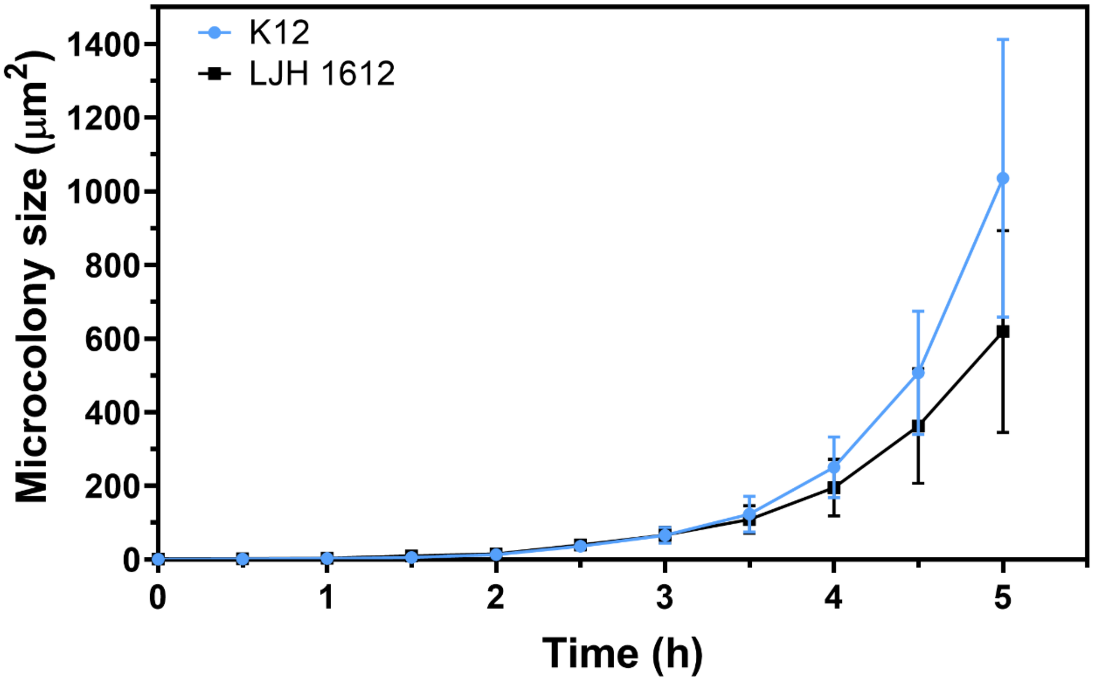

Figure S1. Microcolony size of two representative *E. coli* strains (K12 and LJH 1612) as the function of incubation time. The results were obtained from 100 images for each time point. Error bars represent the standard deviations.

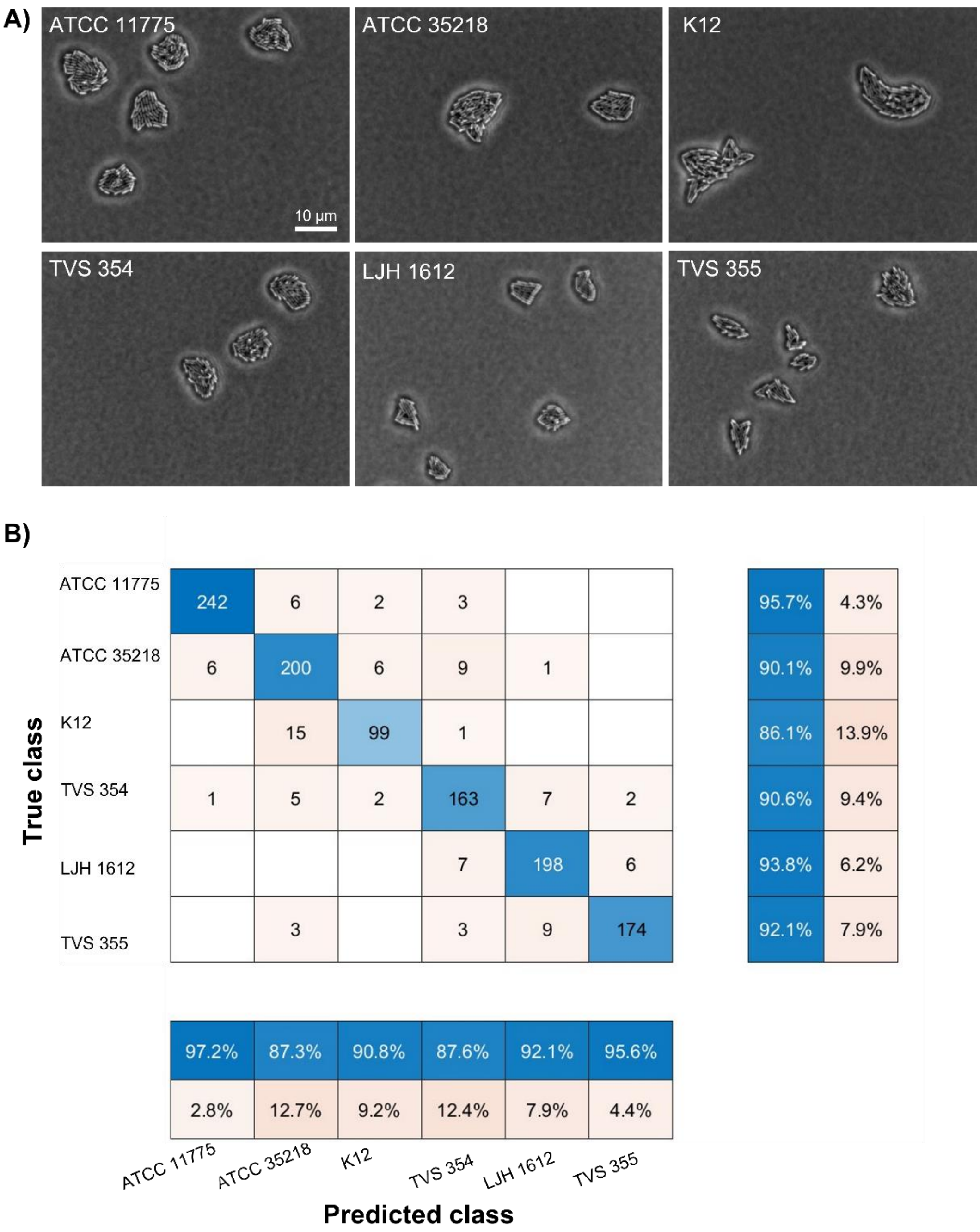

Figure S2. Strain-level discrimination of *E. coli*. (A) Representative images of *E. coli* strains from different isolation sources, including ATCC 11775 (urine), ATCC 35218 (dog), K12 (environment), TVS 354 (lettuce), LJH 1612 (irrigation water), and TVS 355 (soil). (B) Confusion matrix for microcolony classification of *E. coli* strains. For each strain, 315 images were used for machine learning training (60%), validation (10%), and testing (30%).

Tables S1. List of bacterial strains tested in this study.

Bacterial strains		Isolation source	References
Escherichia coli	LJH 1612	Irrigation water	This study
	K12	Animal	[1]
	TVS 354	Lettuce	[2]
	TVS 355	Sandy loam soil	[2]
	ATCC 11775	Urine	[3]
	ATCC 35218	Dog	[4]
Salmonella Enteritidis	PT30	Almond outbreak	[5]
Salmonella Typhimurium	LJH 1689	Snack outbreak	This study
Pseudomonas fluorescens	ATCC 13525	Pre-filtration tanks	[6]
Bacillus subtilis	ATCC 23857	Soil	[7]
Bacillus coagulans	NN0001	Environment	This study
Listeria monocytogenes	LIS0110	Cantaloupe outbreak	[8]
Listeria innocua	ATCC 33090	Cow	[9]

Table S2. Average precision of each class obtained from YOLOv4

Class (bacterial species)	Average precision
Eco (*E. coli*)	0.93
Bco (*Bacillus coagulans*)	0.99
Bsu (*Bacillus subtilis*)	0.94
Lin (*Listeria innocua*)	0.87
Lm (*Listeria monocytogenes*)	0.95
Pfl (*Pseudomonas fluorescens*)	0.96
SE (*Salmonella* Enteritidis)	0.96
ST (*Salmonella* Typhimurium)	0.88
**Mean average precision**	**0.94**

REFERENCES

1. Dimitrova, D., Engelbrecht, K. C., Putonti, C., Koenig, D. W. & Wolfe, A. J. Draft Genome Sequence of Escherichia coli K-12 (ATCC 10798). *Genome Announc.* **5**, e00573-17 (2017).
2. Tomás-Callejas, A. *et al.* Survival and distribution of Escherichia coli on diverse fresh-cut baby leafy greens under preharvest through postharvest conditions. *Int. J. Food Microbiol.* **151**, 216–222 (2011).
3. Wadley, T. D., Jenjaroenpun, P., Wongsurawat, T., Ussery, D. W. & Nookaew, I. Complete genome and plasmid sequences of Escherichia coli type strain ATCC 11775. *Microbiol. Resour. Announc.* **8**, e00046-19 (2019).
4. Angélica Santiesteban-LÓPEZ, N., Rosales, M., Palou, E. & López-Malo, A. Growth response of Escherichia coli ATCC 35218 adapted to several concentrations of sodium benzoate and potassium sorbate. *J. Food Prot.* **72**, 2301–2307 (2009).
5. Isaacs, S. *et al.* An international outbreak of salmonellosis associated with raw almonds contaminated with a rare phage type of Salmonella enteritidis. *J. Food Prot.* **68**, 191–198 (2005).
6. Meier, M. J., Subasinghe, R. M. & Beaudette, L. A. Draft Genome Sequence of the Industrially Significant Bacterium Pseudomonas fluorescens ATCC 13525. *Microbiol. Resour. Announc.* **7**, 4–5 (2018).
7. Wang, C. H., Chang, C. J., Wu, J. J. & Lee, G. Bin. An integrated microfluidic device utilizing vancomycin conjugated magnetic beads and nanogold-labeled specific nucleotide probes for rapid pathogen diagnosis. *Nanomedicine Nanotechnology, Biol. Med.* **10**, 809–818 (2014).
8. Lieberman, V. M. & Harris, L. J. Fate of inoculated Listeria monocytogenes on yellow onions (Allium cepa) under conditions simulating food service and consumer handling and storage. *Food*

Control **96**, 375–382 (2019).

9. Doh, I. J. *et al.* Development of a Smartphone-Integrated Reflective Scatterometer for Bacterial Identification. *Sensors* **22**, 1–12 (2022).
